# Improvement in binge eating and alexithymia predicts weight loss at 9-month follow-up of the lifestyle modification program

**DOI:** 10.1007/s40519-023-01560-5

**Published:** 2023-03-22

**Authors:** Chiara Conti, Maria Di Nardo, Roberta Lanzara, Maria Teresa Guagnano, Valentina Cardi, Piero Porcelli

**Affiliations:** 1 Department of Psychological, Health, and Territorial Sciences, University “G. d’Annunzio” of Chieti-Pescara, Chieti, Italy; 2 Department of Medicine and Aging, University “G. d’Annunzio” Chieti‐Pescara, Chieti, Italy; 3 Section of Eating Disorders, Department of Psychological Medicine, Institute of Psychiatry, Psychology and Neuroscience, King’s College London, London, UK; 4 Department of General Psychology, University of Padova, Padua, Italy

**Keywords:** Obesity, Binge eating, Alexithymia, Self-esteem, Emotional distress, Lifestyle intervention

## Abstract

**Purpose:**

The aim of this longitudinal study was to examine the extent to which improved levels of binge eating (BE) behaviors, alexithymia, self-esteem, and psychological distress would predict a reduction in body mass at 9-month follow-up, following a lifestyle modification program for weight loss in obese or overweight patients.

**Methods:**

A convenience sample of 120 obese or overweight patients were recruited. Body mass index (BMI), binge eating (BES), levels of alexithymia (TAS-20), perceived stress (PSS), depressive symptoms (SDS), and self-esteem (RSE) were assessed during their first medical examination (T1), and after a weight-loss treatment period of 9 months (T2).

**Results:**

Compared with unimproved patients, improved patients reported a significant decrease in binge eating (*p* = 0.04) and perceived stress symptoms (*p* = 0.03), and a significant improvement in self-esteem (*p* = 0.02) over time. After controlling for gender, self-esteem, depressive symptoms, and perceived stress, baseline BMI (OR = 1.11, 95% CI  [1.04,1.19]), ΔBES (OR = 0.99, 95% CI  [0.98,0.99]), and ΔTAS-20 (OR = 1.03, 95% CI  [1.01,1.05]) significantly and independently predicted a ≥ 5% reduction in body mass from baseline.

**Conclusions:**

Our finding supports the suggestion to consider psychological outcomes such as emotional aspects and dysfunctional eating behaviors when planning a weight loss programs to prevent a negative outcome.

Level of evidence: Level III, case–control analytic study.

**Supplementary Information:**

The online version contains supplementary material available at 10.1007/s40519-023-01560-5.

## Introduction

Obesity is a public health disease with widespread medical, psychological, and social implications [1]. Given the obesity epidemic worldwide, significant interventions have been developed for its management. Lifestyle modification, including dieting and exercise, represents one of the core recommended interventions to achieve weight loss and target morbid obesity, overweight and related comorbidities [2]. However, up to 86% of the patients undergoing lifestyle modification do not achieve clinically significant weight loss [3] that is, as indicated by the National Heart, Lung, and Blood Institute [4], losses of 5–10% of initial weight. This can result in weight-regain, relapse from weight loss and its associated comorbidities, and a decrease in quality of life [5]. Thus, identifying predictors of long-term successful weight control is especially important to guide treatment development and identify patients who need increased support or an alternative approach for weight loss [6].

Clinical and sub-clinical eating behaviors, such as binge eating (BE), are usual in subjects seeking weight loss interventions and have been proposed as important mechanisms involved in weight-related changes [7]. Binging behavior, defined by the incorporation of large amounts of food that is accompanied by a sense of loss of control and overeating [8], has been successfully defined in scientific literature. Specifically, BE rates in overweight and obese individuals are at least twice as high as in normal-weight subjects [9], and BE occurs in about 30% of overweight or obese subjects seeking weight loss treatment [10]. In addition, more than 65% of subjects with Binge Eating Disorder (BED) are obese [11], and more than 25% of individuals seeking treatment for obesity fulfill the criteria for BED consistent with DSM-5 [12]. The high prevalence of BE in overweight and obese subjects is alarming, and it might clarify some of the reduced advantages from weight loss treatment, including greater attrition, more subjective barriers, less weight loss, and greater weight regain [12]. Furthermore, weight-related difficulties are associated with psychological problems including psychological distress, depression, and poor self-esteem [13]. Self-esteem and psychological distress typically are included in studies examining the outcomes of weight-related difficulties interventions as it is unclear how they are differentially associated with the outcomes of these interventions [14]. On the other hand, when weight loss programs work, then they might have a positive impact on psychological functioning. For example, in a meta-analysis of psychological outcomes of weight loss interventions [15], authors noted a consistent and significant decrease in depressive symptoms and an improvement in self-esteem following the intervention. However, there is insufficient evidence to assess the impact of weight loss programs on BE at intervention-end [14]. This highlights the importance of assessing multiple psychological processes when evaluating treatment outcomes, both in terms of treatment mediators and in terms of concurrent primary outcomes [16].

Alexithymia is a multifaceted personality dimension widely observed in patients with obesity, especially those with comorbid BE [17, 18]. It is composed of two higher‐order factors: a deficit of affect awareness (as difficulty identifying feelings [DIF] and difficulty describing feelings [DDF]) and operatory thinking (externally‐oriented thinking [EOT] and poor imaginal processes) [19]. These features are assumed to reflect the cognitive deficits in processing and regulation of emotions related to anxiety and depression, and to influence health-related behaviors and symptom onset [20]. High alexithymia has been identified among the risk factors for the onset of several psychological and physical health issues (for a review, see [19]), including reduced weight management, sedentary lifestyle [21], and disordered eating behavior [22]. It has been hypothesized that the alexithymic difficulty in the cognitive processing of emotions induces an amplification of somatic sensations associated with emotional arousal [23]. This could explain the tendency to regulate tension through uncontrolled behaviors, such as BE [24]. Furthermore, individuals with obesity who show higher alexithymic difficulties in identifying and describing their feeling states, are less likely to complete the treatment program and obtain benefits from it. Alexithymia significantly predicts attrition, and unsuccessful weight-loss in obese outpatients [25] and predicts poor treatment outcomes following group cognitive behavioral therapy for overweight and obese patients [26].

This longitudinal study examined the extent to which BE, alexithymia, self-esteem, and psychological distress predicted reduction of body mass (≥ 5% reduction in body mass from baseline) at 9-month follow-up, following a lifestyle modification program for weight loss in obese or overweight patients. The aim of the present study was twofold: (a) to investigate whether BE, alexithymia, distress, and self-esteem would differ between patients not reaching the weight loss threshold (unimproved group) and patients reaching a reduction of ≥ 5% in body mass compared to baseline (improved group); and (b) to explore whether and to what extent changes over time in BE and alexithymia would be associated with treatment outcome (weight loss). Based on previous findings, it was hypothesized that: (a) unimproved patients would exhibit higher levels of BE, alexithymia, and psychological distress, and lower self-esteem than improved patients both at baseline and follow-up; and (b) changes in BE and alexithymia levels over the course of treatment would predict treatment outcome (weight loss) at 9-month follow-up.

## Materials and methods

### Participants and procedure

A sample of 120 treatment-seeking obese and overweight outpatients were enrolled at the Clinical Nutrition Centre of the University Clinical Hospital of Chieti (Italy), consecutively selected from referrals to a dietary control program for any medical reason. Data were collected from April 2017 to December 2018. Patients were evaluated during their first medical examination (T1), and after a weight-loss treatment period of 9 months (T2). All the participants were involved in a non-surgical weight loss program, which was aimed at dietary change, weight control, balanced daily food intake, paced eating, and healthy lifestyle (see Weight loss program section).

To maximize ecological validity, patients aged 18 to 65 and with body mass index (BMI) ≥ 25 were included. Participants were selected for inclusion only if they were enrolled in a weight loss program which did not include the use of drugs. Documented current or past diagnosis of schizophrenia or other psychotic disorders, cognitive impairment, pregnancy, severe medical comorbidity (e.g., thyroid dysfunction, diabetes, chronic liver disease, and any other physical diseases which could interfere with eating behavior), or inability to perform/understand the self‐rating scales were considered exclusion criteria. Patients were evaluated for medical history and past or current psychopathology by a team of expert physicians and psychologists.

### Weight loss program

Standard lifestyle recommendations were provided in written format during weekly 20–30-min individual sessions. Each session was led by a physician and a nutritionist and focused on a discussion around the implementation of a healthy lifestyle and included weight and metabolic monitoring. In addition, patients met a nutritionist once every 3 months until the weight loss goal was achieved. Patients were encouraged to reduce their weight (healthy low-calorie, low-fat diet) and increase their physical activity (moderate-intensity activity, such as walking for at least 150 min per week) following the Food Guide Pyramid [27] and National Cholesterol Education Program guidelines [28].

### Measures

#### Sociodemographic and clinical characteristics

An ad hoc semi-structured questionnaire was used to collect information on sociodemographic characteristics, such as age, gender, marital status, and socioeconomic status (SES). The relationship between educational attainment and job position was used to determine SES [29]. Patient medical records were used to calculated BMI and collect information on the weight history (i.e., years from the first weight-loss treatment). The cutoff of BMI ≥ 25 was used to determine overweight, analysing the ratio of weight in kilograms to the square of height in meters (kg/m^2^).

#### Binge-eating behavior

The severity of BE behavior was measured using the 16‐item Binge Eating Scale (BES) [30]. The BES was originally developed to assess affective, cognitive, and behavioral aspects of BE symptoms in patients with obesity. Scores range from 0 to 46, with a score of ≥ 27 have conventionally served as threshold for severe BE, ≥ 18 for moderate BE, and ≤ 17 for minimal or no BE. Within this sample, Cronbach’s α was 0.86 at T1 and 0.89 at T2.

#### Alexithymia

Levels of alexithymia were measured using the 20-item Toronto Alexithymia Scale (TAS-20) [31]. Each item is scored on a 5-point Likert scale ranging from 1 (= strongly disagree) to 5 (= strongly agree). Scores range from 20 to 100 with a score of ≥ 61 used as the threshold for high alexithymic traits. In addition to the total score, the TAS-20 yields scores for three-factor scales: (1) DIF, a measure of the difficulty to discriminate between feelings and bodily sensations of emotional arousal; (2) DDF, a measure of the difficulty to describe feelings to other people; and 3) EOT, a measure of the tendency to focus on concrete and factual details of external reality and to avoid emotional nuances of emotional life. Within this sample, Cronbach’s α was 0.74 at T1 and0.73 at T2 for the total scale.

#### Self-esteem

Self-esteem was measured using the 10-item Rosenberg Self-Esteem Scale (RSE) [32]. Each item is scored on a 4-point Likert scale ranging from 0 (= strongly disagree) to 3 (= strongly agree). Scores range from 0 to 30 with higher scores indicating a stronger sense of self-esteem. Within this sample, Cronbach’s α was 0.81 at T1 and 0.83 at T2.

#### Depressive symptoms

The Zung Self-Rating Depression Scale (SDS) [33] is a 20-item self-report scale that is used widely to evaluate the severity of psychological and somatic depressive symptoms. The scale was developed based on factor-analytic studies of major depressive disorder as defined in the DSM series and includes all the current DSM-5-TR [8] criteria. Each item is scored on a 5-point Likert scale ranging from 1 (= none or a little of the time) to 4 (= all or almost all the time). Scores range from 20 to 80 with higher scores indicating more severe depressive symptoms. SDS scores are classified as normal (< 50), mild depression (50–59), moderate-to-marked major depression (60–69), and severe-to-extreme major depression (> 70). Within this sample, Cronbach’s α was 0.81 at T1 and 0.80 at T2.

#### Perceived stress

The Perceived Stress Scale (PSS) [34] is a 14-item self-report scale that measures stress from a psychological perspective. Each item is scored on a 5-point Likert scale ranging from 0 (= never) to 4 (= very often). Scores range from 0 to 56 with higher scores indicating a higher level of perceived stress. Within this sample, Cronbach’s α was 0.78 at T1 and 0.84 at T2.

#### Weight outcome measure

Although treatment goals for obesity are numerous, weight loss is the most important. The National Heart, Lung, and Blood Institute [4] panel of experts has indicated the goal of weight loss therapy is to reduce body mass by 5–10% of baseline. While this weight may still be in the overweight or obese range, this modest weight loss can decrease the risk factors for chronic diseases related to obesity [35]. In the present study, the cut-point of 5% of weight loss was used as a cut-point to categorize patients into improved and unimproved outcome groups. Weight data were obtained during the first medical examination (baseline, T1) and after a weight-loss treatment period of 9 months (follow-up, T2).

Non-surgical weight loss is generally reported as a percentage of the initial weight, with a metric called percentage total weight loss (%TWL), expressed as the proportion of change from pre- (T1) to post-treatment (T2) and calculated as follows:

$$\% {\text{TWL}} = \left[ {\left( {{\text{initial weight}} - {\text{current weight}}} \right)/\left( {\text{initial weight}} \right)} \right] \times {1}00.$$

### Statistical analyses

Data analysis was performed using SPSS 26.0 for Windows. Descriptive statistics were reported in terms of mean and standard deviation [Mean (SD)] or absolute frequencies. The level of significance was set at 95%. Alpha for all tests was set at 0.05, with all *p* values being adjusted for multiple comparisons with the false discovery rate method, using the Benjamini–Hochberg procedure [36].

A three-step strategy was used for data analysis.

First, independent and paired-sample Student’s *t* tests or chi-square tests (*χ*^2^) were used to compare between- and within-group differences in socio-demographic and clinical variables over time for improved and unimproved patients. Cohen’s *d* and Cramer’s *φ* were used as measures of effect size.

Second, repeated-measures analysis of covariance (ANCOVA) was used to compare between-group differences in psychological variables that are based on repeated observations while controlling for a confounding variable. The repeated-measures ANCOVA included BE, levels of alexithymia, self-esteem, depressive symptoms, and perceived stress as dependent variables, the timepoints T1 and T2 as a within-subject factor, BMI at baseline as a covariate, and TWL%-related groups as the between-subject factor. The partial eta-squared (*η*^2^) was used as a measure of effect size.

Third, binary logistic regression analysis was used to investigate how change in psychological variables (i.e., BE, levels of alexithymia, self-esteem, depressive symptoms, and perceived stress) predicted the weight outcome. The measurement of change over time (Δ) in psychological variables, expressed as the proportion of change from T1 to T2, was calculated as follows: [(T2–T1)/(T1)] × 100. The weight outcome was considered as a dependent variable (dummy coded: 0 = unimprovement; 1 = improvement). The independent variables were gender, baseline BMI, changes in BE, levels of alexithymia, self-esteem, depressive symptoms, and perceived stress. Six regression steps were processed and regression coefficients, confidence intervals (CI), odds ratio (OR), and *p* values were estimated. In the first step, gender and baseline BMI were entered as control variables. In the next steps, we added the variables that we were interested in. Specifically, to evaluate the contribution of BE and alexithymia to the outcome before adjusting for other clinical variables, ΔBES and ΔTAS-20 were included in the second and third steps. In the following steps, we added other key variables (ΔRSE in the fourth step, ΔSDS in the fifth step, and ΔPSS in the sixth step). In particular, we aimed to investigate the extent to which each factor would significantly distinguish between the two outcome groups.

## Results

### Characteristics of the sample

**Fig. 1:**
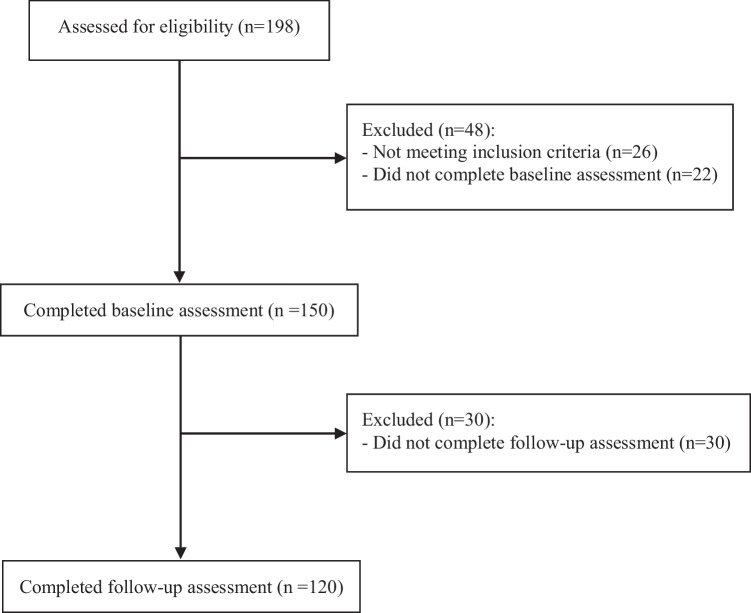
Consort diagram describing the flow of participation in the study (created using MS Office)

Figure 1 describes the flow of participation in the study. One hundred and ninety-eight participants were screened for eligibility. One hundred and fifty (75.6%) were eligible and participated in the study. Of the 150 participants assessed at T1, 30 (20%) were lost at follow-up and did not complete the measures at T2, and 120 (80%) were included in the present study. No baseline differences were found between patients who completed and those who did not completed the follow-up (Table S1).

Table 1 reports the socio‐demographic and clinical characteristics of the sample.

**Table 1:** Socio‐demographic and clinical characteristics of the study sample (*N* = 120)

Variable	Total sample *N* = 120	Improved group *N* = 63 (52.5%)	Unimproved group *N* = 57 (47.5%)	*t*/*χ*^2^	*p*	*d*/*φ*
Age, mean (SD)	48.92 (14.20)	48.03 (13.87)	50.11 (14.64)	0.79	0.420	0.15
Gender						
Men	41 (34.2%)	27 (65.9%)	14 (34.1%)	4.45	0.031	0.19
Women	79 (65.8%)	36 (45.6%)	43 (54.4%)			
SES						
Middle-low	65 (52.4%)	35 (53.9%)	30 (46.1%)	0.68	0.412	0.08
Middle-high	55 (47.6%)	28 (50.9%)	27 (49.1%)			
Marital status						
Unmarried	60 (50%)	20 (48.4%)	21 (51.6%)	0.04	0.830	0.02
Married	60 (50%)	43 (50.7%)	42 (49.3%)			
History of overweight (years), mean (SD)	7.61 (9.23)	6.62 (8.91)	9.03 (9.68)	1.06	0.295	0.26
BMI (T1), mean (SD)	37.58 (8.02)	39.69 (7.89)	35.39 (7.72)	2.99	0.003	0.56
BMI (T2), mean (SD)	35.22 (7.58)	35.56 (7.51)	34.72 (7.61)	0.59	0.550	0.12

*SES*  socioeconomic status

Included patients were mostly females (*n* = 79, 65.8%), with a mean age of 48.92 years (SD = 14.20 years) and had been overweight for 7.61 years (SD = 9.23 years). Most of the participants were from a middle-low socioeconomic status (*n* = 65, 52.4%) and 50% were married (*n* = 60). According to the NHLBI criteria (see Methods section), 52.5% (*n* = 63) of the sample achieved the 5% weight loss criterion at T2. Compared with unimproved, improved patients were predominantly men (*n* = 27, 65.9%; *φ* = 0.19) and had a higher baseline BMI (*d* = 0.56). No differences were found on age, SES, marital status, and history of overweight between the two groups.

### Within‑group comparisons over time

Table 2 reports the differences in clinical variables between baseline (T1) and follow-up (T2) in the total sample.

**Table 2:** Comparisons of BMI, binge eating (BES), alexithymia (TAS-20), self-esteem (RES), depressive symptoms (SDS), and psychological distress (PSS) before (T1) and after (T2) treatment in total sample

Variable	Total sample (*N* = 120)		*t*	*p*’	*d*
	T1 Mean (SD)	T2 Mean (SD)			
BMI	37.58 (8.02)	35.22 (7.58)	6.44	0.002	0.30
BES	10.34 (7.63)	7.91 (8.89)	3.56	0.002	0.29
TAS-20	47.63 (12.77)	45.41 (12.59)	2.64	0.003	0.18
TAS-DIF	15.63 (6.72)	14.38 (6.39)	2.91	0.009	0.19
TAS-DDF	13.04 (5.25)	12.27 (5)	1.97	0.064	0.15
TAS-EOT	18.96 (4.54)	18.76 (5.21)	0.48	0.630	0.04
RSE	19.11 (4.41)	20.08 (4.66)	2.61	0.018	0.21
SDS	46.51 (10.33)	45.91 (11.21)	0.72	0.528	0.04
PSS	23.05 (7.26)	21.62 (8.92)	2.14	0.045	0.18

*p*’ = Benjamini–Hochberg adjusted *p* value

*BES*  Binge Eating Scale, *TAS-20*  Toronto Alexithymia Scale–20, *TAS-DIF*  Toronto Alexithymia Scale–Difficulty Identifying Feelings, *TAS-DDF*  Toronto Alexithymia Scale–Difficulty Describing Feelings, *TAS-EOT*  Toronto Alexithymia Scale–Externally Oriented Thinking, *RSE*  Rosenberg Self-Esteem Scale, *SDS*  Zung Self-Rating Depression Scale, *PSS*  Perceived Stress Scale

All patients reported overall improvement as compared to before treatment. Statistically significant T1 vs. T2 differences were found for BMI (*d* = 0.30), BES (*d* = 0.29), TAS-20 (*d* = 0.18) and its affective factor DIF (*d* = 0.19), RSE (*d* = 0.21), and PSS (*d* = 0.18) (all small effect sizes).

### Between‑group comparisons over time

Table S2 (online appendix) describes the differences in clinical and psychological variables at baseline (T1) and follow-up (T2) between improved and unimproved groups. When the two groups were compared at baseline, unimproved patients had significantly higher levels of alexithymia (*d* = 0.45) and depressive symptoms (*d* = 0.47) compared to improved patients (all moderate effect sizes). When the two groups were compared at follow-up, unimproved patients had higher BES (*d* = 0.44), PSS (*d* = 0.58), SDS (*d* = 0.65), and lower RSE (*d* = 0.60) (all moderate effect sizes).

Table 3 reports the results of repeated measure ANCOVA. The baseline level of BMI was used as a covariate to investigate its influence on patients’ weight loss at follow-up.

**Table 3:** Comparisons of psychological variables before (T1) and after (T2) treatment between improved and unimproved groups

Variable mean (SD)	TWL%-related groups			Time			Time*BMI(T1)			Time*Group	
	Improved *N* = 60	Unimproved *N* = 56	*F*	*p*’	η^2^	*F*	*p*’	*η*^2^	*F*	*p*’	*η*^2^
BES											
T1	9.88 (6.95)	10.82 (8.34)	2.50	0.190	0.02	0.30	0.601	0.00	4.12	0.050	0.03
T2	6.25 (6.42)	9.18 (7.97)									
TAS-20											
T1	45.38 (12.34)	50.75 (12.78)	0.15	0.690	0.00	0.92	0.425	0.00	1.64	0.245	0.01
T2	44 (12.19)	47.52 (12.92)									
RSE											
T1	19.55 (4.08)	18.39 (4.73)	4.31	0.140	0.04	2.35	0.350	0.02	5.45	0.050	0.04
T2	21.35 (4.21)	18.70 (4.84)									
SDS											
T1	44.43 (9.74)	48.70 (10.73)	0.72	0.490	0.00	1	0.425	0.01	1.15	0.276	0.01
T2	42.60 (10.04)	49.41 (11.54)									
PSS											
T1	22.20 (6.76)	24.18 (7.611)	3.64	0.140	0.03	2.17	0.350	0.02	4.91	0.050	0.04
T2	19.42 (7.56)	23.91 (9.38)									

*p*’ = Benjamini–Hochberg adjusted *p* value

*BES* Binge Eating Scale, *TAS-20*  Toronto Alexithymia Scale–20, *RSE*  Rosenberg Self-Esteem Scale, *SDS*  Zung Self-Rating Depression Scale, *PSS*  Perceived Stress Scale

Both time and baseline BMI covariate did not show a significant effect on any variables. Compared with unimproved patients, improved patients reported a significant decrease in BES (*F* = 4.12, *p*’ = 0.050) and PSS (*F* = 4.91, *p*’ = 0.050), and a significant improvement in RSE (*F* = 5.45, *p*’ = 0.050) over time with effect sizes in the small range.

### Predicting weight outcome

Table 4 shows binary logistic regression model with the %TWL score as a binary outcome criterion (improvement/unimprovement). Gender, baseline BMI, and changes (Δ) in psychological features served as independent variables.

**Table 4:** Logistic regression model examining changes in psychological variables from baseline to end of treatment as predictors of treatment outcomes at 9-month follow-up

Variables	Step 1		Step 2		Step 3		Step 4		Step 5		Step 6	
	*β*	OR [95% CI]	*β*	OR [95% CI]	*β*	OR [95% CI]	*β*	OR [95% CI]	*β*	OR [95% CI]	*β*	OR [95% CI]
Gender	1.92	1.80 [0.78, 4.16]	2.19	1.96 [0.80, 4.76]	2.69	2.14 [0.86, 5.29]	2.74	0.97 [0.85–1.12]	3.21	2.37 [0.92, 6.07]	3.33	2.41 [0.94, 6.22]
BMI (T1)	4.89*	1.06 [1, 1.13]	7.67**	1.03 [0.80, 4.76]	7.85**	1.09 [1.03, 1.17]	8.50**	1.06 [0.93–1.22]	8.13**	1.11 [1.03, 1.17]	8.54**	1.11 [1.04, 1.19]
ΔBES			9.46**	0.99 [0.98, 0.99]	10.91**	0.99 [0.98, 0.99]	9.91**	0.93 [0.82–1.06]	10.07**	0.99 [0.98, 0.99]	9.46**	0.99 [0.98, 0.99]
ΔTAS-20					3.58*	1.02 [1, 1.04]	4.53*	0.95 [0.85–1.05]	5.83**	1.03 [1.01, 1.05]	6.17**	1.03 [1.01, 1.05]
ΔRSE							2.61	1.10 [0.86–1.42]	1.88	1.01 [0.99, 1.03]	1.98	1.01 [0.99, 1.03]
ΔSDS									2.43	0.98 [0.95, 1]	1.57	0.98 [0.96, 1.01]
ΔPSS											0.80	0.99 [0.98, 1.01]
*R*^2^		0.11		0.23		0.27		0.29		0.32		0.33
Δ*R*^2^		0.11		0.12		0.04		0.02		0.03		0.01
*χ*^2^		9.39**		11.31**		3.88*		2.81		2.61		0.80

*BES*  Binge Eating Scale, *TAS-20*  Toronto Alexithymia Scale–20, *RSE*  Rosenberg Self-Esteem Scale, *SDS*  Zung Self-Rating Depression Scale, *PSS*  Perceived Stress Scale

^*^p < 0.05; **p < 0.01; ***p < 0.001

In the first model, gender and baseline BMI explained 11% of %TWL, with only baseline BMI showing the greatest OR of 1.06 (95%CI  [1, 1.13]). Adding ΔBES (OR = 0.99, 95%CI [0.98, 0.99]) produced an added prediction of 12% (Model 2). When ΔTAS-20 was added in Model 3, it significantly predicted an added 4% of the variance with a significant OR of 1.02 (95%CI  [1, 1.04]). Adding ΔRSE, ΔSDS, and ΔPSS (Models 4, 5, and 6) did not contribute to explain a significant added variance. The final model (predictive accuracy = 72.7%) showed that baseline BMI (OR = 1.11, 95%CI  [1.04, 1.19]), ΔBES (OR = 0.99, 95%CI  [0.98, 0.99]), and ΔTAS-20 (OR = 1.03, 95% CI  [1.01, 1.05]) significantly and independently predicted %TWL.

## Discussion

The aim of this longitudinal study was to examine the extent to which BE, alexithymia, self-esteem, and psychological distress would predict a reduction of body mass at 9-month follow-up, following a lifestyle modification program for weight loss in obese or overweight patients. The main result suggests that improvement in BE and alexithymia may play a clinically significant role in predicting a decrease in body mass at 9-month follow-up. In particular, after controlling for gender, self-esteem, depressive symptoms, and perceived stress, overweight and obese patients who did not benefit from weight loss control program were more alexithymic and had more BE behaviors. Our finding is consistent with a growing number of studies that have established that an improvement in psychological outcomes is associated with a reduction of body mass after a lifestyle modification program for weight loss [15, 37]. In addition, this result is in line with literature that has revealed a mechanism based on emotional dysregulation and uncontrolled eating underlying problems related to body mass [38]. This supports the recommendation to include psychological outcomes such as emotional aspects and dysfunctional eating behaviors when designing a weight loss program to prevent a negative outcome.

In our first hypothesis, we expected that unimproved patients would report higher levels of BE, alexithymia, and psychological distress, and lower self-esteem than improved patients both at baseline and follow-up. This hypothesis was partially confirmed. At baseline, patients who did not improve had significantly higher levels of alexithymia and depressive symptoms compared to improved patients. This result is in line with evidence suggesting depressive symptoms and difficulties in identifying and describing feelings as risk factors for negative weight loss program outcomes. The finding that depressive symptoms are associated with negative weight loss program outcomes is well-established [37, 39]. Depressive symptoms and emotional regulation strategies may predict negative weight loss program outcomes and, in turn, negative treatment outcomes might predict the onset and the maintenance of psychological distress [17, 37, 39]. For example, functional emotion regulation strategies, like reappraisal, predicted weight loss in a sample of adolescents undergoing inpatient obesity treatment [40]. Alexithymic personality traits are frequently observed in people with obesity and eating disorders [17, 18] and individuals with obesity or overweight who show higher difficulties in identifying and describing their feeling states, are less likely to complete the treatment program and obtain benefits from it [25, 26]. This is in line with evidence suggesting that individuals with high levels of alexithymia may be less likely to obtain benefits from treatment due to difficulties in building collaborative therapeutic relationships [40], limited adaptive coping in stressful situations [19], associations with unhealthy behaviors [41]. Furthermore, it is possible that difficulties to discriminate affective states from bodily feelings, and hunger from satiety could have a negative impact on adherence to dietetic recommendations [12]. Indeed, neuroimaging evidence show that alexithymia is associated with reduced neural responses to emotional stimuli from the external environment, and with enhanced neural activity in somatosensory and sensorimotor regions [42]. Deficits in the cognitive processing and regulation of emotions are associated with depression and may reinforce each other. This may further contribute to affect health-related behaviors, symptom formation, and numerous mental and physical health issues (for a review, see [19]).

In this study, 52.5% of the patients achieved the 5% weight loss criterion at a 9-month follow-up. This result is in line with the literature showing a percentage of successful participants ranging from 14.7% to 67% in short-to-medium-term lifestyle interventions [3]. When the two groups of improved and unimproved patients were compared at follow-up, those who had benefited more reported a significant decrease in BE and perceived stress and depressive symptoms, and a significant improvement in self-esteem following treatment. In fact, when weight loss programs work, then the weight loss might have a positive impact on psychological functioning [15]. For example, recent studies indicate that weight loss due to caloric restriction or gastric bypass surgery improves depressive symptoms among obese patients with depression [43, 44].

In our second hypothesis, we expected that changes in BE and alexithymia over the course of treatment would predict treatment outcomes. This hypothesis was confirmed. Baseline BMI and improvement in BE and alexithymia predicted successful outcome, over and above changes in self-esteem and psychological distress. Instead, in our sample no significant changes in depressive symptoms were reported at follow-up. Indeed, change in depressive symptoms did not predict treatment outcome. The result that BE symptoms and alexithymia levels predicted successful outcome is coherent with evidence that psychological treatments targeting emotions and problematic eating behaviors, often result in a significant increase in the efficacy of therapeutic weight loss interventions [38]. Indeed, overweight and obese patients with comorbid BE, tend to have lower levels of emotional awareness and difficulty in using emotion regulation strategies (e.g., [45]) and less favorable prognosis compared to those without BE (e.g., [46]). Some hypotheses to explain how alexithymic difficulties in affective awareness are associated with the key mechanisms in the onset and maintenance of BE and to explain how these mechanisms contribute to the health and weight issues in obese and overweight patients have been established. For example, it seems that individuals with higher alexithymic characteristics abuse external regulators, such as food for regulating emotional arousal [47], being unable to cognitively process their emotions adaptively [48]. Our results are in line with studies that have shown a mechanism based on emotional dysregulation and bingeing behaviors underlying issues related to body mass and suggest that clinicians certainly need to identify and monitor alexithymia and BE in their patients, and include them among their therapeutic interventions strategies for reducing alexithymia and BE and for mitigating their effects on the health and weight issues.

### Strength and limits

The longitudinal study design, the high response rate, and the use of well-validated questionnaires are some of the major strengths of this study. However, there are also some limitations to acknowledge. First, the naturalistic design and the lack of a control group limit the extent to which causal conclusions can be drawn about the relationship between BE, alexithymia, self-esteem, psychological distress, and body mass reduction in patients who engage in a lifestyle modification program. Second, a consecutive non‐probabilistic sample was used in this study, which may have hindered our findings’ validity due to the risk of selection bias. For example, participants were selected from a specific clinical site and results might not generalize to patients attending different interventions. In addition, this study is based on a substantial percentage of individuals who volunteered for weight loss treatments, thus limiting the generalizability to patients who do not seek treatment. Third, psychological variables were assessed only with self-report. Self-reported measures involve a significant amount of personal insight and can be influenced by subjective biases and social desirability effects. Finally, several potential predictors were not controlled for, so 27% of the variance in weight loss was explained by other factors. There were several unmeasured variables (e.g., smoking habit, lack of exercise, alcohol abuse, and personality disorders) that could help us further understand the relationship between psychological factors and weight loss achievement. Therefore, future research will benefit from exploring other factors as potential influences on lifestyle modification programs.

To conclude, with these limitations in mind, results from the current study indicate that overweight and obese patients who present higher BE, and, mainly, alexithymic difficulties in the cognitive processing of emotions, are less likely to gain advantages from the treatment program. This shows the significance of evaluating BE and alexithymic deficits when assessing treatment outcomes, both in terms of treatment mediators and in terms of concomitant primary outcomes. Therefore, the results of the current study emphasize the importance of recognizing individuals with BE and alexithymic deficits before the start of treatment and during weight loss intervention to address psychological treatments on these outcomes. Tailored treatments on outcomes such as the decrease of BE and difficulties in the cognitive processing of emotions should be prudently assessed by clinicians to improve the therapeutic effectiveness of weight control program.

## Supplementary Information

Below is the link to the electronic supplementary material.

Supplementary file1 (DOCX 20 KB)

## Data Availability

The data sets generated during and/or analysed during the current study are available from the corresponding author on reasonable request.
