# Supplementary material for: Improvement in binge eating and alexithymia predicts weight loss at 9-month follow-up of the lifestyle modification program
Source: Eat Weight Disord. 2023 Mar 22;28(1):30. doi: 10.1007/s40519-023-01560-5 (PMC10033561; doi:10.1007/s40519-023-01560-5)
Supplement: Supplementary file 1 — Supplementary file1 (DOCX 20 KB) [file 40519_2023_1560_MOESM1_ESM.docx]

**Eating and Weight Disorders - Studies on Anorexia, Bulimia and Obesity**

**Improvement in binge eating and alexithymia predicts weight loss at nine-month follow-up of the lifestyle modification program**

Chiara Conti, Maria Di Nardo, Roberta Lanzara*, Maria Teresa Guagnano, Valentina Cardi, Piero Porcelli

***Correspondence:** Roberta Lanzara, Department of Psychological, Health, and Territorial Sciences, University “G. d’Annunzio” of Chieti-Pescara, Chieti, Italy. ORCID: 0000-0001-8159-5218. E-mail: roberta.lanzara@uniroma1.it

**Additional Information**

**TABLE S1.** Socio‐demographic and clinical characteristics for those who completed and those who did not complete the follow-up (N = 150).

| **Variable** | **Total sample**  **N = 150** | **Completed**  **N = 120 (80%)** | **Not completed**  **N = 30 (20%)** | **t/χ^2^** | **p** | **d/φ** |
| --- | --- | --- | --- | --- | --- | --- |
| Age, *mean (SD)* | 47.40 (14.07) | 48.92 (14.20) | 46 (13.42) | 1.04 | .572 | 0.21 |
| Gender |  |  |  |  |  |  |
| Male | 48 (32%) | 41 (34.2%) | 7 (23.3%) | 1.29 | .280 | 0.09 |
| Female | 102 (68%) | 79 (65.8%) | 23 (76.7%) |  |  |  |
| SES |  |  |  |  |  |  |
| Middle-low | 81 (54%) | 65 (52.4%) | 16 (53.3%) | 0.24 | .670 | 0.04 |
| Middle-high | 69 (46%) | 55 (47.6%) | 14 (46.7%) |  |  |  |
| Marital status |  |  |  |  |  |  |
| Unmarried | 77 (51.3%) | 60 (50%) | 17 (56.6%) | 0.44 | .801 | 0.06 |
| Married | 73 (48.7%) | 60 (50%) | 13 (43.4%) |  |  |  |
| History of overweight  (years), *mean (SD)* | 8.04 (9.71) | 7.61 (9.23) | 9.57 (11.39) | 0.25 | .810 | 0.20 |
| BMI (T1), *mean (SD)* | 37.40 (7.62) | 37.58 (8.02) | 36.41 (5.54) | 0.07 | .792 | 0.16 |

Note: SES = Socioeconomic Status

**TABLE S2.** Comparisons of BMI, binge eating (BES), alexithymia (TAS-20), self-esteem (RES), depressive symptoms (SDS), and psychological distress (PSS) before (T1) and after (T2) treatment in the improved and unimproved groups.

| **Variable** | **Improved group (N = 63)**  *Mean (SD)* | **Unimproved group (N = 57)**  *Mean (SD)* | **t** | **p’** | **d** |
| --- | --- | --- | --- | --- | --- |
| BES (T1) | 9.78 (6.95) | 10.96 (8.34) | 0.84 | .400 | 0.16 |
| BES (T2) | 6.11 (6.34) | 9.19 (7.90) | 2.36 | .033 | 0.44 |
| TAS-20 (T1) | 45.08 (12.22) | 50.44 (12.88) | 2.33 | .033 | 0.45 |
| TAS-20 (T2) | 43.44 (12.16) | 47.58 (12.81) | 1.81 | .100 | 0.33 |
| RSE (T1) | 19.71 (4.07) | 18.44 (4.70) | 1.59 | .122 | 0.29 |
| RSE (T2) | 21.38 (4.14) | 18.65 (4.81) | 3.34 | <.001 | 0.60 |
| SDS (T1) | 44.06 (9.71) | 48.75 (10.64) | 2.51 | .025 | 0.47 |
| SDS (T2) | 42.56 (9.96) | 49.49 (11.45) | 3.51 | <.001 | 0.65 |
| PSS (T1) | 21.95 (6.85) | 24.26 (7.57) | 1.75 | .100 | 0.32 |
| PSS (T2) | 19.27 (7.62) | 24.21 (9.57) | 3.14 | .006 | 0.58 |

p’=Benjamini-Hochberg adjusted p-value

Note: BES = Binge Eating Scale; TAS-20 = Toronto Alexithymia Scale – 20; RSE = Rosenberg Self-Esteem Scale; SDS = Zung Self-Rating Depression Scale; PSS = Perceived Stress Scale.
